# Design, Analysis and Experiment of a Tactile Force Sensor for Underwater Dexterous Hand Intelligent Grasping

**DOI:** 10.3390/s18082427

**Published:** 2018-07-26

**Authors:** Jianjun Zhang, Weidong Liu, Li’e Gao, Yiwen Zhang, Weijiang Tang

**Affiliations:** 1 School of Marine Science and Technology, Northwestern Polytechnical University, Xi’an 710072, China; sitanjj@mail.nwpu.edu.cn (J.Z.); gao_li_e@nwpu.edu.cn (L.G.); weijiangtang2018@gmail.com (W.T.); 2 Science and Technology on Underwater Information and Control Laboratory, Northwestern Polytechnical University, Xi’an 710072, China; 3 The 705 Research Institute, China Shipbuilding Industry Corporation, Xi’an 710077, China; zhangyiwen1805@gmail.com

**Keywords:** underwater tactile force sensor, water pressure compensation, silicon cup, Finite Element Analysis, experiment

## Abstract

This paper proposes a novel underwater dexterous hand structure whose fingertip is equipped with underwater tactile force sensor (UTFS) array to realize the grasping sample location determination and force perception. The measurement structure, theoretical analysis, prototype development and experimental verification of the UTFS are purposefully studied in order to achieve accurate measurement under huge water pressure influence. The UTFS is designed as capsule shape type with differential pressure structure, and the external water pressure signal is separately transmitted to the silicon cup bottom which is considered to be an elastomer with four strain elements distribution through the upper and lower flexible contacts and the silicone oil filled in the upper and lower cavities of UTFS. The external tactile force information can be obtained by the vector superposition between the upper and lower of silicon cup bottom to counteract the water pressure influence. The analytical solution of deformation and stress of the bottom of the square silicon cup bottom is analyzed with the use of elasticity and shell theory, and compared with the Finite Element Analysis results, which provides theoretical support for the distribution design of four strain elements at the bottom of the silicon cup. At last, the UTFS zero drift experiment without force applying under different water depths, the output of the standard force applying under different water depth and the test of the standard force applying under conditions of different 0 ${}^{\circ }$C–30 ${}^{\circ }$C temperature with 0.1 m water depth are carried out to verify the performance of the sensor. The experiments show that the UTFS has a high linearity and sensitivity, and which has a regular zero drift and temperature drift which can be eliminated by calibration algorithm.

## 1. Introduction

**Figure 1:**
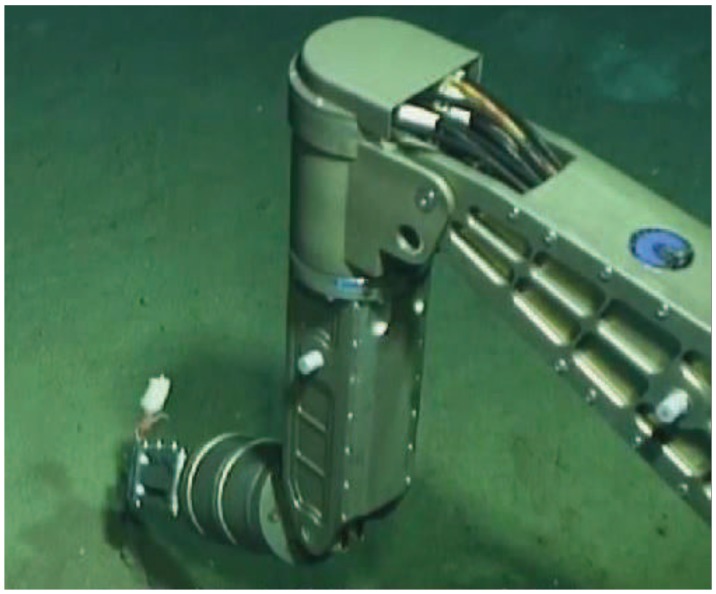
The task diagram of underwater manipulator without force sensor.

The underwater manipulator is an indispensable part of the underwater vehicle, performing the tasks such as biological samples collection, underwater salvage and so on [1,2,3]. However, due to the huge water pressure, uncertain operation object and lack of force perception, it will bring over operation or miss operation to limit the task efficiency [4,5]. The underwater sight of the task area is easily influenced by the rotation of the propeller, which disturbs the observation of the underwater camera, and the underwater manipulator will perform local search if force measurement acquired [6]. Nearly all underwater manipulators of current generation, which are controlled by the handle operation with camera observe, are not equipped with UTFS. As shown in Figure 1, the pilots manipulate the manipulator to implement the sample collection; however, it may miss or hurt the sample, and easily fail to grasp the moving creature without the tactile force measurement. As a result, the development of the underwater dexterous hand incorporating force or torque sensing is a direction for the underwater intelligent grasping. The basic feature of an underwater dexterous hand is to imitate human finger skin, consisting of high density force perception components to sense external force information. The tactile sensor array is distributed on the surface of the finger, and the position determination and tactile force measurement of the grasping target are realized, so that the tactile force sensor is the core of the measurement.

Recently, many scholars have proposed various types of two-dimensional or multi-dimensional force sensors based on different principles that convert the mechanical deformation to the electrical signal [7,8,9,10]. The typical measurement method is with the use of the resistance strain whose component consistent of elastic structure body and semiconductor sensitive element with the Micro-Electro-Mechanical System (MEMS) development [11]. As a result, there exist lots of force sensors in the non-underwater environment [12,13], however, the sensors employed in the underwater environment with few species have to be redesigned differently with the reason of high water pressure and wide measurement range. Additionally, the salinity and density of seawater bring anticorrosion, sealing and insulation problems to UTFS design, and the water fluid resistance and inertial force bring disturbance to the measurement of UTFS. Therefore, it is important to study the measurement principle and structure design for the UTFS of underwater dexterous hand array sensor to achieve intelligent grasping under complex environment.

Different underwater tactile force measurement approaches and the structure design have been studied for many years. The basic measurement principle is to measure the elastomer strain information caused by the tactile force under the condition that the external water pressure is balanced on the surface of the elastomer. The most important and reliable underwater tactile sensor is a multi-dimensional force-torque sensor with beam structure elastomer [7,8,9,10], whose force feedback output is abundant without static water pressure disturbance and the sealing problems [11]; however, it cannot determine the location of tactile force and the complex elastic shape of parallel mechanical structure may cause the cross sensitivity problem. The tactile sensor based on the strain gauge has realized the tactile force measurement under the water pressure balance [14,15], but each strain gauge needs to correspond to a Wheatstone bridge [16], which causes the problem of complex circuit, additionally the strain gauge’s paste process has an impact on signal output. Paper [17,18] proposed optoelectronic type force sensor and Paper [19,20] introduced fiber bragg grating (FBG) force sensor, while the light attenuation may affect the output with long time use and the sensor light is susceptible to the external cover light.

The tactile force sensor can be used for material recognition [21], object shape perception [22] and pose estimation [23] in addition to tactile perception.The optoelectronic device [24] and fiber optics tactile array probe [25] are also important tactile measurement equipment. Kampmann et al. [26,27] designed an underwater dexterous hand, where the piezoelectric sensors and fiber optic sensor array are mounted on the end-effector with a planar surface, and the joint connection are equipped with the force-torque sensors in order to acquire the tactile information as much as possible. There is an indirect sensing method to measure the liquid pressure or the electric current information for the hydraulically actuated manipulator [28,29] or motor-actuated underwater manipulator [30] avoiding water pressure disturbance, but hardly obtain the true force feedback value. Qiaokang Liang designed an underwater manipulator prototype with a novel 4-D fingertip force sensor with an E-type membrane [31,32], and the prototype has abundant force/torque information output with good linearity and high sensitivity. In paper [33], a conceptual design of a novel tactile sensor with capacitive transduction, and used as artificial skin for a deep-sea manipulator is presented. In [34], the tactile sensing for an underwater operation system based on multi-finger sensors information fusion is presented, and the strain-gauges are used to achieve force measurement. D.J.O.Brien and D.M.Lane designed and employed a dexterous hand named AMADEUS [35,36], and force and slip information were gained with strain gauge force sensor and PVDF (Polyvinylidene Fluoride) piezoelectric film-based vibration sensor, while the whole structure couldn’t judge the slip directions in the single fingertip and ignored the temperature influence. Much work such as structure design, temperature compensation, zero-point compensation and calibration equipment design can be done in order to realize the accuracy and stability output of underwater tactile force measurement under water pressure disturbance.

To accurately determine the grasping position and the force feedback value measurement of the underwater manipulator sample collection process under the influence of water static pressure, we propose a structure of an underwater dexterous hand with an array of UTFSs for each fingertip. The distribution of tactile force sensor array on the finger of dexterous hand is purposefully introduced to determine the position of grasping sample, and the tactile force sensor which makes up the sensor array is studied emphatically. Consequently the force sensing principle, the structure design, assembly and experimental verification of tactile force sensor are purposefully studied. The position distribution theory of four strain elements on the surface of silicon cup elastomer is analyzed, and Finite Element Analysis is implemented for the sensor’s high resolution and high sensitivity output. This paper is organized as follows: In Section 2 the dexterous hand structure and the UTFS structure with differential pressure are designed. In Section 3 the strain and stress of the silicon-cup bottom elastomer under uniform pressure is analyzed for the four strain elements distribution on elastomer surface. In Section 4 the Finite Element Analysis is employed for the silicon-cup bottom under uniform pressure is achieved and compared with analytical solution method; In Section 5 the analysis of the silicon cup measurement with linear output is carried out; In Section 6 the experiment is done for the designed UTFS verification and discuss the experiment and conclude the paper in Section 7 and Section 8, respectively.

## 2. The Structures of Dexterous Hand and UTFS

**Figure 2:**
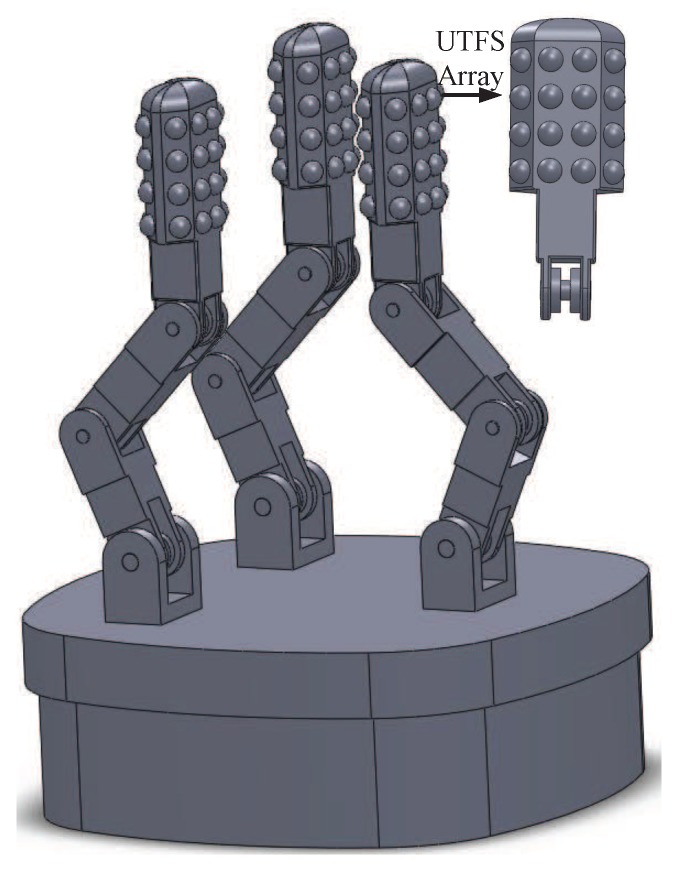
The structure of underwater dexterous hand with UTFS array.

The detail of the dexterous hand structure is shown in Figure 2, according to the human fingertip structure and the principle of sensory sensation of human skin. The dexterous hand is designed with three fingers, and each finger contains three degree of freedom (DOF) because dexterous hand mainly depends on the function of pinching and grasping to realize underwater sample collection. Each finger is driven by a servo motor which is mounted in the palm of the hand and connected to the finger joints, and the fingers grasping and releasing motion of the manipulator is achieved through the forward and reverse direction rotation of the servo motor. A low density UTFS array is mounted on the front and side views of the Distal Interphalangeal Point (DIP) joint of each finger for the dexterous hand intelligent grasping. The UTFS array consists of two kinds of force sensors, and one of which composed of eight force sensors can measure the force information of the fingertip inner surface. The other kind of UTFS can obtain the information of grasping force on the side, and the left and right side are composed of four force sensors respectively. The force measurement principle is that the upper hemispherical flexible contact as cover transfers the external water pressure and tactile force information to silicon cup elastomer through silicon oil, at the same time, the low hemispherical flexible contact also as cover transfers the external water pressure to the other side of the silicon cup elastomer through silicon oil to balance the water pressure, which makes the silicon cup elastomer directly measure the external tactile force signal by water pressure vector superposition. The screw holes are machined in the bottom of the dexterous hand to connect with the robot arm.

The ideal UTFS can be considered as a part of the dexterous hand finger different from the commercially available sensor core, because the silicon-cup elastomer is directly mounted in the machining hole of the fingertip, and the inner wall of the machining hole is directly used as the shell of UTFS. What is more, the flexible contact is connected with the machining hole through the thread and the seal ring, so that the design method is beneficial to reduce the volume of the finger and more UTFSs may be distributed on the fingertip. However, with the reason of the processing technology and cost requirements, the actual developed UTFS has its own shell and commercial silicon-cup sensor core to achieve basic verification.

**Figure 3:**
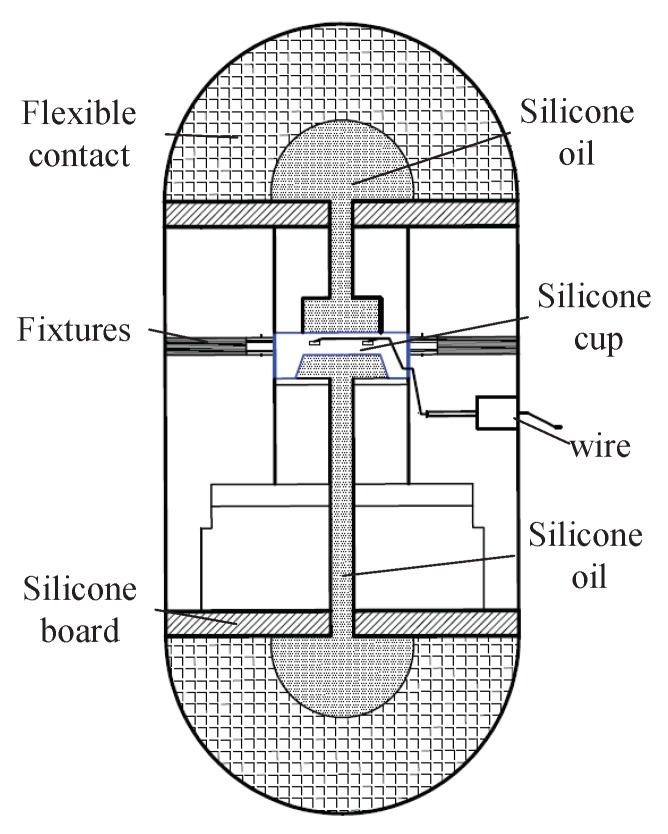
The structure diagram of capsule UTFS.

According to the measurement requirement of the UTFS under complex underwater environment, a capsule-shaped UTFS including two flexible contacts is designed referring to principle of strain piezoresistive silicon sensor measuring liquid pressure. The structure diagram of the UTFS is shown in Figure 3, and the UTFS consists of flexible contacts, silicone oil, fixture, silicon cup, wire, silicon board, etc. The UTFS displays the capsule shape, and the silicon-cup type elastomer is embedded in the fixing device, which divides the UTFS into upper and lower parts containing a cavity filled with silicone oil separately. The upper and lower part respectively has a spherical flexible contact which can sense the external force and isolates the external water environment and the internal silicone oil environment. The flexible contact is made of the mixture of Nitrile Butadiene Rubber (NBR) and silica gel, which is soft and keeps the hemisphere shape easily to contact with the external target, and can transmit the external contact deformation information to the internal silicone oil pressure information without any loss. Through the internal silicone oil, the pressure information is transmitted to the upper and lower sides of the silicon-cup bottom elastomer so as to realize the purpose of sensing tactile force information under the static pressure vector overlay of the water.

Therefore, the differential pressure capsule-shaped UTFS transforms the external tactile force into the pressure of silicone oil inside the sensor, and the silicone oil pressure is uniformly loaded on the silicon cup bottom elastomer containing four strain elements to deform, so that the four strained elements constituting the full bridge are realized to the tactile force measurement. The fabrication process of silicon cup and the distribution of strain element in the bottom of the silicon cup play an important role in the nonlinearity and sensitivity of the signal output.

## 3. Mathematical Model of the Force Analysis of Silicon Cup Bottom

The core of the silicon cup bottom elastomer composed of four strain elements is the main component of force measurement technique. The fabrication process of silicon cup elastomer is to sputter the protective film (such as $\mathrm{Si}O_{2}$ and ${\mathrm{Si}}_{3}N_{4}$) on the front surface of the monocrystalline silicon [37], and the position of the strain element is determined by the lithography technology of the protective film, then the vacuum sputtering force sensitive material protection film (such as Ti) and the force sensitive material (such as Au) are realized, and the production of four strain elements is achieved by stripping and cleaning the force-sensitive material [38]. The positive cavity of cup body is formed by lithography and anisotropic etching with corrosive liquid (such as KOH) on the bottom of monocrystalline silicon, and the whole silicon cup elastomer system of silicon cup is completed. The four strain elements on the silicone elastomer are led out to the outside through a wire to form a Wheatstone Bridge, and the silicone elastomer and the protective shell form the core of the sensor core, which can be purchased on the commercial market. The pressure measurement is achieved by the linear relationship between the strain and the output of Wheatstone Bridge, so that the strain and stress analysis at the bottom of the silicon-cup elastomer is the core of the whole measurement.

The silicon cup bottom elastomer containing four strain elements is subjected to the pressure of the top side and the bottom side, and which can be regarded as a square thin plate with four sides fixed and a thickness smaller than the side length. The stress and strain distribution of the square thin plate under uniform pressure loading can be analyzed by elasticity and plate shell theory, which is of theory support for the arrangement of the strain element on the high stress region of the square thin plate to achieve high resolution and sensitivity signal output.

### 3.1. Mathematical Model of Silicon Cup Bottom

**Figure 4:**
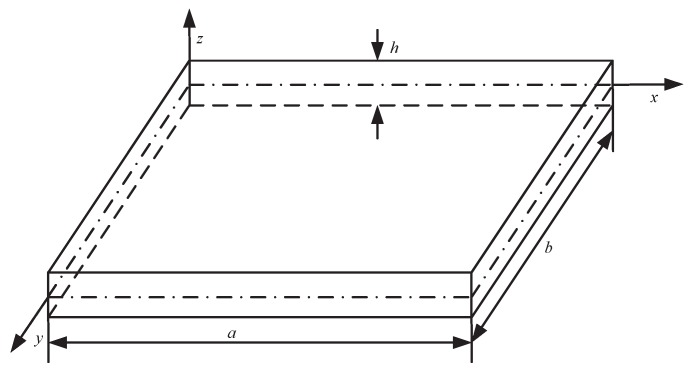
The square diaphragm with four fixed sides.

Suppose the square thin plate size as a×b×h, which is shown in Figure 4. The deflection is much smaller than the diaphragm thickness, which satisfies the Kirchhoff hypothesis so that the square thin plate can be considered to satisfy the small deflection theory under the uniform pressure $p_{0}$. Combined with the thin plate bending theory, the stress component of any point in the thin plate can be expressed by the thin plate deflection with the equilibrium differential equation of elasticity, geometric equation and physical equation [39]. The bending stress and shear stress of any point in the thin plate are illustrated as:

(1) $$\begin{array}{c} \left\{\begin{array}{c} \sigma_{x}=-\frac{Ez}{1-\mu^{2}}\left(\frac{\partial^{2}w}{\partial x^{2}}+\mu \frac{\partial^{2}w}{\partial y^{2}}\right)\\ \sigma_{y}=-\frac{Ez}{1-\mu^{2}}\left(\frac{\partial^{2}w}{\partial y^{2}}+\mu \frac{\partial^{2}w}{\partial x^{2}}\right)\\ \tau_{xy}=-\frac{Ez}{1+\mu }\frac{\partial^{2}w}{\partial x\partial y} \end{array}\right. \end{array}$$

where *w* represents the deflection of any point in the thin plate, and *z* represents the ordinate of the xOy surface. $\sigma_{x}$, $\sigma_{y}$, $\tau_{xy}$ represent the transverse stress, the longitudinal stress and shear stress, respectively. *E* and μ represent elastic modulus and Poisson ratio of the thin plate, respectively.

### 3.2. Stress Analysis of the Square Thin Plate

The deflection and stress distribution of the thin plate are analyzed by the Ritz method, which is an approximate analysis method based on the principle of minimum potential energy [40]. The basic principle is to take the finite term of the series expansion of displacement, and to make the infinite number of position parameters become finite polynomial as well as to apply the stationary condition of potential energy according to the principle of minimum potential energy. To solve the deflection *w* of the four-side fixed square plate under uniform load $p_{0}$ by the method of the Ritz, with Figure 4 the boundary condition of the thin plate is expressed as:

(2) $$\begin{array}{c} \left\{\begin{array}{cc} w{|}_{x=0}=0; & w{|}_{y=0}=0;\\ w{|}_{x=a}=0; & w{|}_{y=b}=0;\\ \frac{\partial w}{\partial x}{|}_{x=0}=0; & \frac{\partial w}{\partial y}{|}_{y=0}=0;\\ \frac{\partial w}{\partial x}{|}_{x=a}=0; & \frac{\partial w}{\partial y}{|}_{y=b}=0; \end{array}\right. \end{array}$$

An infinite degree of freedom system is replaced by a finite degree of freedom system, and the approximate solution is solved according to the principle of minimum potential. For the square thin plate shown in Figure 4, the triple triangular series expansion of any point of deflection satisfying the boundary condition is shown in Equation (3):

(3) $$\begin{array}{c} \begin{array}{c} w=\sum_{m=1}^{\infty }\sum_{n=1}^{\infty }K_{mn}(1-\cos\frac{2m\pi x}{a})\left(1-\cos\frac{2n\pi y}{b}\right) \end{array} \end{array}$$

where *a* and *b* are the length and width of the square thin plate respectively, and (x,y) represents the coordinate of any point on the thin plate with the range of 0≤x≤a and 0≤y≤b, and $K_{mn}$ is the triple triggering coefficient expansion coefficient independent of *x* and *y* under the uniform load $p_{0}$. It is obvious that each item of the series in Equation (3) can satisfy the fixed boundary condition in Equation (2), and the elastic deformation energy of the plate can be expressed as:

(4) $$\begin{array}{c} \begin{array}{cc} U & =\frac{D}{2}\underset{A}{\;\int \int \;}{\left(\frac{\partial^{2}w}{\partial x^{2}}+\frac{\partial^{2}w}{\partial y^{2}}\right)}^{2}dxdy\\ & =\frac{D}{2}\int_{0}^{a}\int_{0}^{b}\left\{\sum_{m=1}^{\infty }\sum_{n=1}^{\infty }4\pi^{2}K_{mn}\left[\frac{m^{2}}{a^{2}}\cos\frac{2m\pi x}{a}(1-\right.\right.{\left.\left.\cos\frac{2n\pi y}{b})+\frac{n^{2}}{b^{2}}\cos\frac{2n\pi y}{b}\left(1-\cos\frac{2m\pi x}{a}\right)\right]\right\}}^{2}dxdy\\ & =2D\pi^{4}ab\left\{\sum_{m=1}^{\infty }\sum_{n=1}^{\infty }\left[3\left(\frac{m^{4}}{a^{4}}\right)+3\left(\frac{n^{4}}{b^{4}}\right)+2\left(\frac{m^{2}}{a^{2}}\right)\left(\frac{n^{2}}{b^{2}}\right)\right]K_{mn}^{2}\right.\\ & \left.+\sum_{m=1}^{\infty }\sum_{r=1}^{\infty }\sum_{s=1,r\neq s}^{\infty }2(\frac{m^{4}}{a^{4}})K_{mr}K_{ms}+\sum_{r=1}^{\infty }\sum_{s=1,r\neq s}^{\infty }\sum_{n=1}^{\infty }2(\frac{n^{4}}{b^{4}})K_{rn}K_{sn}\right\} \end{array} \end{array}$$

where *A* represents the thin plane area of the plate, and *D* represents the bending stiffness of the thin plate [41] which can be expressed as

(5) $$\begin{array}{c} D=\frac{Ez^{3}}{12(1-\mu^{2})} \end{array}$$

where *z* is the thin plate thickness.Under the action of uniform load $p_{0}$, the external potential energy *V* can be expressed as:

(6) $$\begin{array}{c} \begin{array}{cc} V & =-\int_{0}^{a}\int_{0}^{b}p_{0}\sum_{m=1}^{\infty }\sum_{n=1}^{\infty }K_{mn}(1-\cos\frac{2m\pi x}{a})\left(1-\cos\frac{2n\pi y}{b}\right)dxdy\\ & =-p_{0}ab\sum_{m=1}^{\infty }\sum_{n=1}^{\infty }K_{mn} \end{array} \end{array}$$

The total potential energy Π with Equations (4) and (6) can be expressed as: Π=U+V. By the minimum potential energy principle the choice of $K_{mn}$ should meet $\partial \Pi /\partial K_{mn}=0$, according to Π’s taking the extreme conditions it can be obtained:

(7) $$\begin{array}{c} \begin{array}{c} \begin{array}{c} 4\pi^{4}Dab\left\{\left[3\left(\frac{m^{4}}{a^{4}}\right)+3\left(\frac{n^{4}}{b^{4}}\right)+2\left(\frac{m^{2}}{a^{2}}\right)\left(\frac{n^{2}}{b^{2}}\right)\right]K_{mn}\right.\\ \left.+\sum_{r=1,r\neq n}^{m}2\left(\frac{m^{4}}{a^{4}}\right)K_{mr}+\sum_{r=1,r\neq m}^{n}2\left(\frac{n^{4}}{b^{4}}\right)K_{rn}\right\}-p_{0}ab=0 \end{array} \end{array} \end{array}$$

where set m=1, n=1 and the first item in Equation (7) can be obtained:

(8) $$\begin{array}{c} \begin{array}{c} K_{11}=\frac{p_{0}a^{4}b^{4}}{4\pi^{4}D\left(3b^{4}+3a^{4}+2a^{2}b^{2}\right)} \end{array} \end{array}$$

Substituting Equation (8) with Equation (3) and the deflection of the plate is obtained with the first term of the series expressed as:

(9) $$\begin{array}{c} \begin{array}{c} w=\frac{p_{0}a^{4}b^{4}\left(1-\cos\frac{2\pi x}{a}\right)\left(1-\cos\frac{2\pi y}{b}\right)}{4\pi^{4}D\left(3b^{4}+3a^{4}+2a^{2}b^{2}\right)} \end{array} \end{array}$$

where *w* will obviously obtain the maximum value when x=a/2 and y=b/2.

The integral stress distribution at the bottom of silicon cup is analyzed by Von-Mises equivalent stress. The Von-Mises equivalent stress refers to a physical quantity that measures the yield state of a material by comparing the stress combination with the yield limit of the stress state the condition of complex stress and expressed as:

(10) $$\begin{array}{c} \begin{array}{cc} \sigma_{V-M}= & ([{\left(\sigma_{x}-\sigma_{y}\right)}^{2}+{\left(\sigma_{y}-\sigma_{z}\right)}^{2}+{\left(\sigma_{z}-\sigma_{x}\right)}^{2}\\ & +6{\left(\tau_{xy}+\tau_{yz}+\tau_{zx}\right)}^{2}{]/2)}^{\frac{1}{2}} \end{array} \end{array}$$

where $\sigma_{x}$, $\sigma_{y}$ and $\sigma_{z}$ are corresponding to the stress in the direction of the *x*, *y*, *z* axis respectively, and $\tau_{xy}$, $\tau_{yz}$ and $\tau_{zx}$ are corresponding to the shear stress on the xOy, yOz and zOx surface respectively. According to the characteristics of the thin plate, making $\sigma_{z}=\tau_{yz}=\tau_{zx}=0$ and substituting Equation (1) with Equation (10) and it can be obtained the Von-Mises equivalent stress which can be expressed as:

(11) $$\begin{array}{c} \begin{array}{cc} \sigma_{V-M}= & \frac{Ez}{1-\mu^{2}}(\left(\mu^{2}-\mu +1\right)\left[{\left(\frac{\partial^{2}w}{\partial x^{2}}\right)}^{2}+{\left(\frac{\partial^{2}w}{\partial y^{2}}\right)}^{2}\right]\\ & +\left(-\mu^{2}+4\mu -1\right)\frac{\partial^{2}w}{\partial x^{2}}\frac{\partial^{2}w}{\partial y^{2}}+3\left(1-\mu^{2}\right){\left(\frac{\partial w}{\partial x}\frac{\partial w}{\partial y}\right)}^{2}{)}^{\frac{1}{2}} \end{array} \end{array}$$

## 4. Finite Element Analysis of Silicon Bottom

The Finite Element Analysis software ANSYS Workbench 14.0 is used to analyze the bottom of the silicon cup under uniform load [42]. Simultaneously, the strain and Von-Mises stress with the Ritz method are calculated and plotted in Matlab software to realize the comparison of the Finite Element Analysis method, and the distribution of stress and strain at the bottom of the silicon cup is obtained.

**Figure 5:**
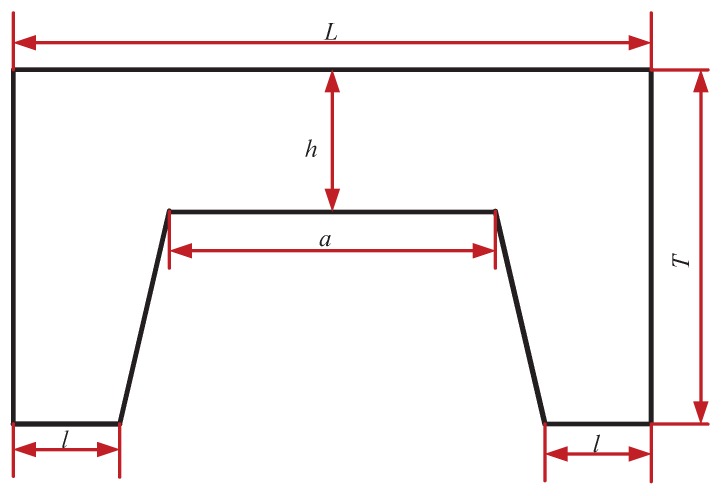
The side view diagram of the silicon cup.

The side view of the silicon cup structure is shown in Figure 5. The width, the edge width and the height of the silicon cup are expressed as: L×L=4.3×4.3 mm, l=1 mm and T=0.39 mm. According to the characteristics of the silicon cup corrosion process, the thickness of the strain diaphragm on the silicon cup is h=0.09 mm. As a result of the bottom of the square, the side length is a=1 mm. It is assumed that the silicon-cup bottom is under uniform pressure and silicon-cup material is of uniform property, and the structure is symmetrical and the mathematical model is established for the whole silicon cup.

**Figure 6:**
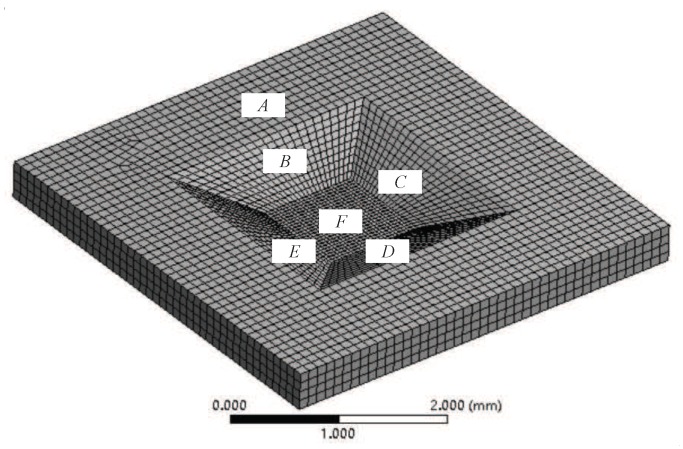
The model mesh of the silicon cup with load and constraint.

The material properties are added to the model of the silicon cup geometry. The elastic modulus is set as 190 GPa and the Poisson ratio is 0.28. Employing Solid186 hexahedral element mesh method and the model is divided into 154,530 nodes and 33,786 units. The model mesh, the constraint and load applied are shown in Figure 6. It is applied fixed constraints in the region of A, B, C, D, E and 1 MPa uniform load is applied at the bottom of the cup in the F region.

**Figure 7:**
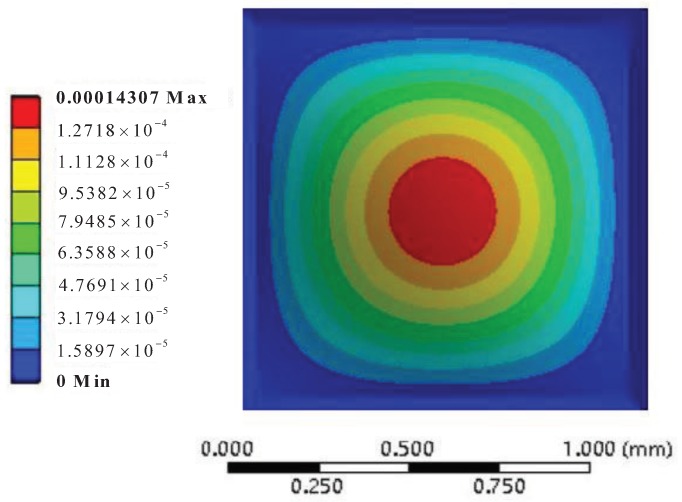
The deformation nephogram of silicon cup with Finite Element Analysis.

**Figure 8:**
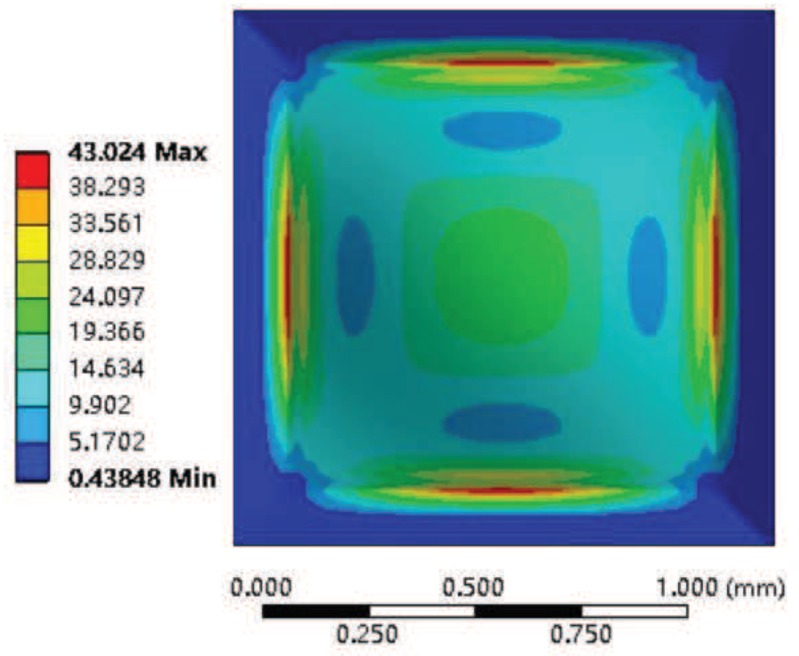
The Von-Mises stress nephogram of silicon cup with Finite Element Analysis.

The deformation nephogram and the Von-Mises equivalent stress nephogram of the silicon cup bottom employing the Finite Element Analysis method are shown in Figure 7 and Figure 8.

**Figure 9:**
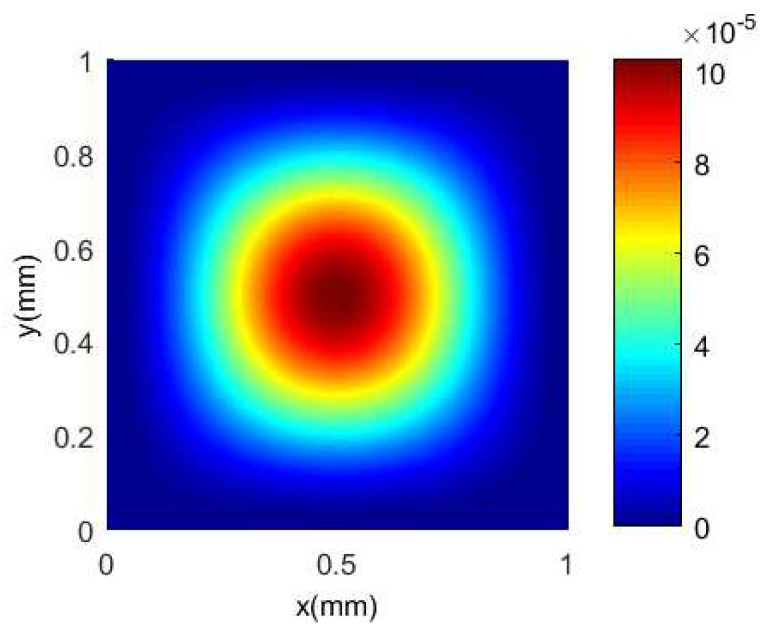
The Matlab nephogram of silicon cup deflection analytical solution.

**Figure 10:**
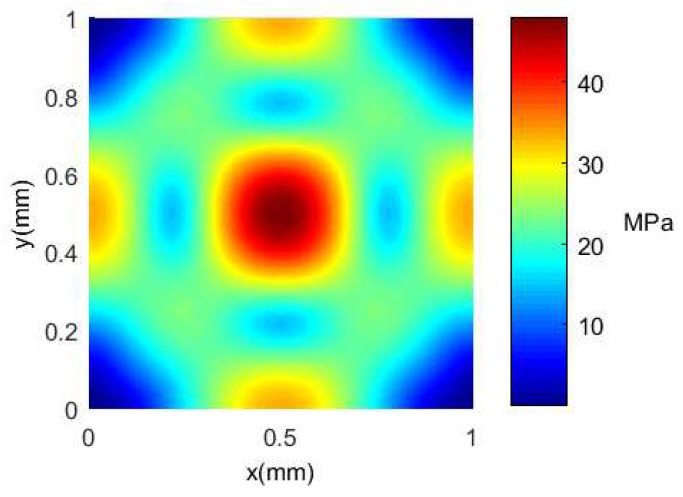
The Matlab nephogram of silicon cup Von-Mises stress analytical solution.

The same with the Finite Element Analysis method, the material properties, the geometric size and the pressure value of the silicon cup bottom are substituted into Equations (9) and (11), and the analytical solution of the deflection and the Von-Mises stress of the silicon cup bottom can be obtained. The analytical solution of the deflection and Von-Mises equivalent stress in the Matlab software is calculated and plotted as shown in Figure 9 and Figure 10.

According to the comparison between Figure 7 and Figure 9, the finite element deformation nephogram of the silicon cup bottom is consistent with that of the analytical solution of Matlab software, and the deflection increases gradually from the surrounding to the center, and the deflection is the largest at the center of the silicon cup bottom [43]. The maximum deflection error of the two methods is within 18%.

According to the comparison between Figure 8 and Figure 10, it is concluded that the finite element Von-Mises equivalent stress nephogram at the silicon cup bottom is consistent with the basic value of the stress analytical solution Matlab nephogram, and the stress at the midpoint of four side and the central position is the largest. The maximum error of the two methods is within 25%.

There are errors between the results of Finite Element Analysis method and Matlab theoretical calculation method, of which the main reason is with different modeling method. The Finite Element Analysis method is based on the real geometry model of silicon cup, and the result is solved by dividing the body unit grid. The Matlab analytical solution method is equivalent to the bottom of the silicon cup as a solid plate model, ignoring the thickness of the shear stress and shear stress. So that the error is reasonable, and the results of the two methods can be verified to each other.

## 5. Analysis of the Silicon Cup Measurement

To obtain the maximum value of the transverse and longitudinal stress difference at the bottom of the silicon cup, the theoretical basis is provided for the distribution of the force sensing element in the bottom of the cup. The term $\sigma_{x}-\sigma_{y}$ is calculated according to Equations (1) and (9) which can be obtained as:

(12) $$\begin{array}{c} \begin{array}{cc} \sigma_{x}-\sigma_{y} & =\frac{-Ez}{1+\mu }\left(\frac{\partial^{2}w}{\partial x^{2}}-\frac{\partial^{2}w}{\partial y^{2}}\right)\\ & =\frac{-3p_{0}a^{2}\left(1-u\right)}{2\pi^{2}z^{2}}\left(\cos\frac{2\pi x}{a}-\cos\frac{2\pi y}{a}\right) \end{array} \end{array}$$

**Figure 11:**
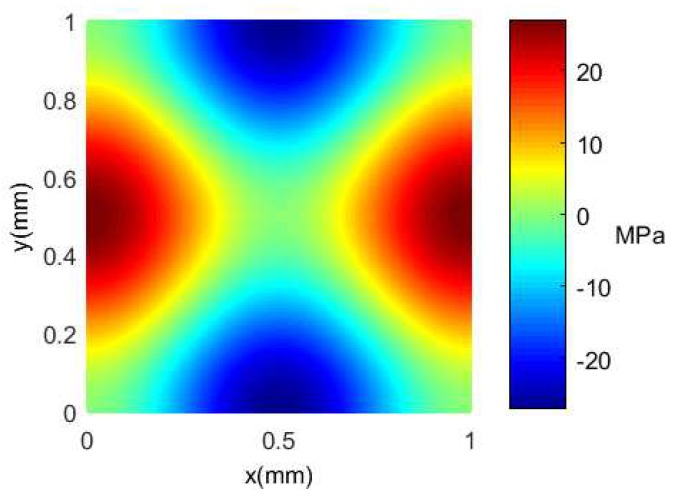
The MATLAB nephogram of $\sigma_{x}-\sigma_{y}$ distribute.

The material properties, the geometric size and the pressure value of the bottom of the silicon cup are substituted into the Equation (12), and the distribution nephogram of the stress difference of the silicon cup bottom can be obtained and shown in Figure 11.

It is concluded from Figure 11 that the midpoint of a pair of edges is subjected to the largest transverse stress, which shows positive strain under the condition of uniform pressure, on the other hand, the longitudinal stress at the midpoint of the other pair is the largest, showing a negative strain, and the maximum and minimum values of $\sigma_{x}-\sigma_{y}$ are respectively 27.019 and −27.019 MPa. If the force sensing element is distributed in the region of high stress at the silicon cup bottom, the output signal with high sensitivity is obtained.

**Figure 12:**
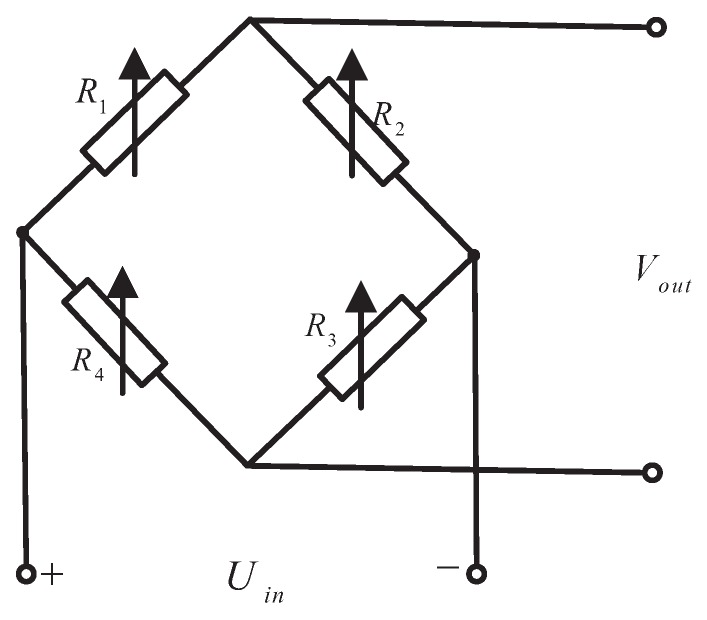
The silicon cup full-bridge circuit with four force-sensitive components.

Referring to the literature [44] the four strain elements mounted in the silicon-cup bottom will constitute the Wheatstone bridge which is shown in Figure 12, according to the piezoresistive effect of silicon chip and suppose Wheatstone full arm bridge resistance as $R_{i}\left(i=1,2,3,4\right)$. When the strain of the strain gauge is achieved, the change rate of resistance value of the bridge arm is expressed as:

(13) $$\begin{array}{c} \begin{array}{c} \left\{\begin{array}{c} \frac{\Delta R_{1}}{R_{1}}=\frac{\Delta R_{3}}{R_{3}}=\frac{\pi_{44}}{2}\left(\sigma_{x}-\sigma_{y}\right)\\ \frac{\Delta R_{2}}{R_{2}}=\frac{\Delta R_{4}}{R_{4}}=\frac{\pi_{44}}{2}\left(\sigma_{y}-\sigma_{x}\right) \end{array}\right. \end{array} \end{array}$$

where $\sigma_{x}$ and $\sigma_{y}$ are transverse and longitudinal stresses respectively, and $\pi_{44}$ is shear piezoresistive coefficient. According to the relationship between the longitudinal and transverse stress distribution in the silicon cup bottom, a pair of opposite sides are mainly subjected to longitudinal stress, and the other pair of edges are mainly subjected to transverse stress, so that the two groups of force sensing element coefficient are opposite. As a result, the four strain elements form a Wheatstone bridge in the full arm bridge performance, enabling high resolution and sensitivity signal measurements, and the signal output has a linear relationship with the applied load [45].

## 6. Experiment and Test of the UTFS

### 6.1. UTFS Assembly

**Figure 13:**
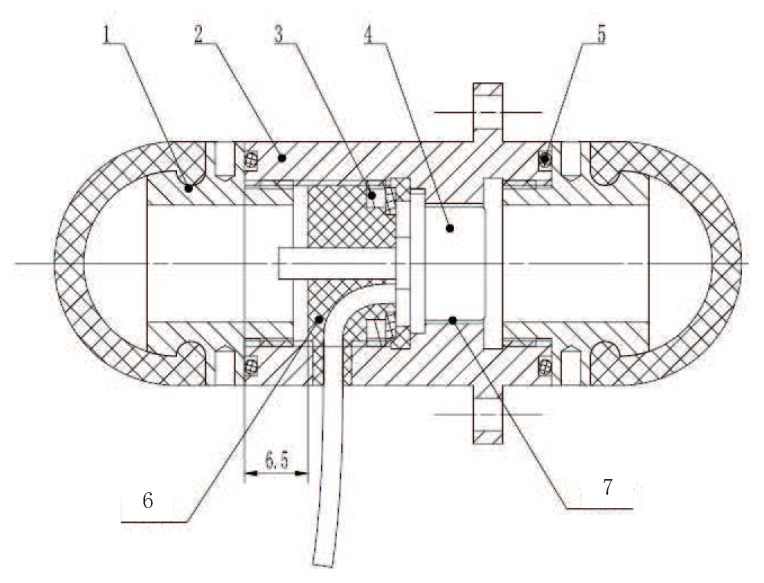
The schematic diagram of underwater UTFS assembly. (Note: 1. Flexible contact; 2. Sensor shell; 3. Snap ring; 4. Sensor core; 5. O sealing ring; 6. Epoxy Resin AB glue; 7. Glue filling cavity).

The processing technology and assembly process of the sensor have an important influence on the output performance of the sensor. The UTFS is designed and manufactured according to the previously described measurement principle of the UTFS, and the designed UTFS profile is shown in Figure 13. Considering the underwater sealing, the power supply and the signal output of the sensor core, the parts of UTFS mainly include the flexible contact, the sealing ring, the shell, the sensor core, snap rings, the Poly Tetra Fluoroethylene (PTFE) pad and so on, which is similar with the previously described principles of the sensor structure in Figure 3. The sensor core whose measurement center is silicon-cup with four strain elements is waterproof proceed, mounted to the sensor housing from the lower end hole. The sensor core circuit board is covered with a PTFE insulation mat to prevent the circuit damage, consequently the sensor core is fixed and pressed with a snap ring. The selected sensor core parameters are shown at Table 1.

The sensor core wire is led to the outside of the sensor shell through the guide hole in the shell, the sensor core hole conducts the lower cavity and the silicon-cup elastomer, which transmits the cavity oil pressure to the surface of the silicon-cup elastomer, and the epoxy AB glue is used to pour sealing and fix the wire, and also to fix the guide hole. The gap between the upper shell of the sensor core and the inner shell of the UTFS is also sealed by epoxy AB glue. The cavity between the end of the sensor core and the flexible contact is filled with silicon oil to transmit the oil pressure to the other surface of the silicon-cup elastomer to construct differential performance.

**Figure 14:**
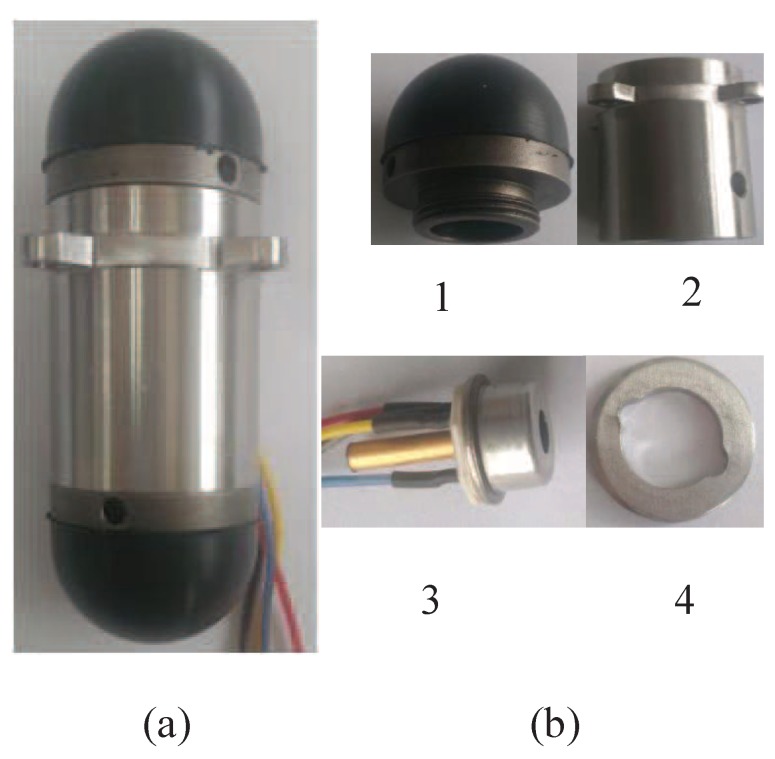
The photo of UTFS and part components. (**a**): UTFS; (**b**): part components (Note: 1. Flexible contact; 2. Sensor shell; 3. Sensor core; 4. Snap ring).

The flexible contact includes the flexible part and the stainless steel metal sleeve, and flexible part consistent of silicone and NBR is vulcanized and adhered to the stainless steel metal sleeve, which has the characteristics of good sealing and corrosion resistance, and the stainless steel metal sleeve is fixed on the shell of the UTFS through the thread. The assembly process of the flexible contacts and the UTFS shell housing is completed in the silicone oil environment, which ensures the silicone oil is fully filled with the upper and lower cavities. The thread between the flexible contact and UTFS shell has a large tolerances, which realizes the silicone oil to discharge to the outside of the housing shell to avoid generating the additional pressure when rotation assembly. The two flexible contacts are equipped with the shell at the same time to keep the up and down cavities pressure equivalent, and the seal ring is employed to prevent silicone oil from escaping. The photo of UTFS and part components are shown in Figure 14. The selected sensor core can be directly purchased on the market, which avoids the production of a sensor core or a strain gauge, ensuring the accuracy requirements and linear output of the UTFS. During the experiment, the Wheatstone bridge of the sensor core is directly powered and the output signal is measurement.

### 6.2. UTFS Zero Point Output at Different Depth

The sensor core of UTFS measurement circuit can be regarded as a full arm bridge performance of Wheatstone bridge [46], and the output signal sensitivity is higher than the single arm, half-bridge signal output. The full bridge circuit is shown in Figure 12, and the measurement signal output is achieved under $U_{in}$ = 7.4 V voltage Power supply.

**Figure 15:**
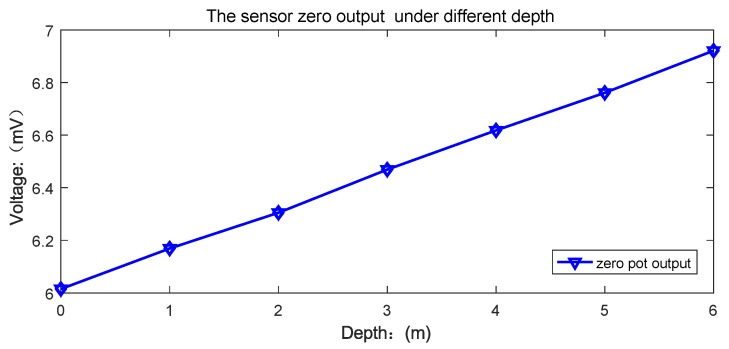
Zero output voltage at different depths.

To detect the water pressure influence to the UTFS output, the zero point output experiment under different depths is realized. The UTFS is placed at different water depth ranging from 0 m to 6 m, and the output of the Wheatstone bridge is measured without any tactile force load. The zero output curves at different water depth are shown in Figure 15, which can be seen that the sensor zero point output increases linearly with water depth, from 6 mV at 0 m water depth to 6.9 mV at 6 m water depth.

The main reason for the zero point drift at different water depth is that the volume of the upper and lower cavities are different and the volume of the filled silicon oil of the two cavities are also different, so that the upper and lower silicon oil pressure are different with the deformation extrusion of the flexible contacts under the effect of equal external water pressure. The installation process is the secondary cause of the UTFS zero point drift. Because of the linear zero point drift, and the output signal influence due to water depth can be diminished with the data fusion calibration algorithm with water depth measurement.

### 6.3. UTFS Experiment at Different Water Depth

**Figure 16:**
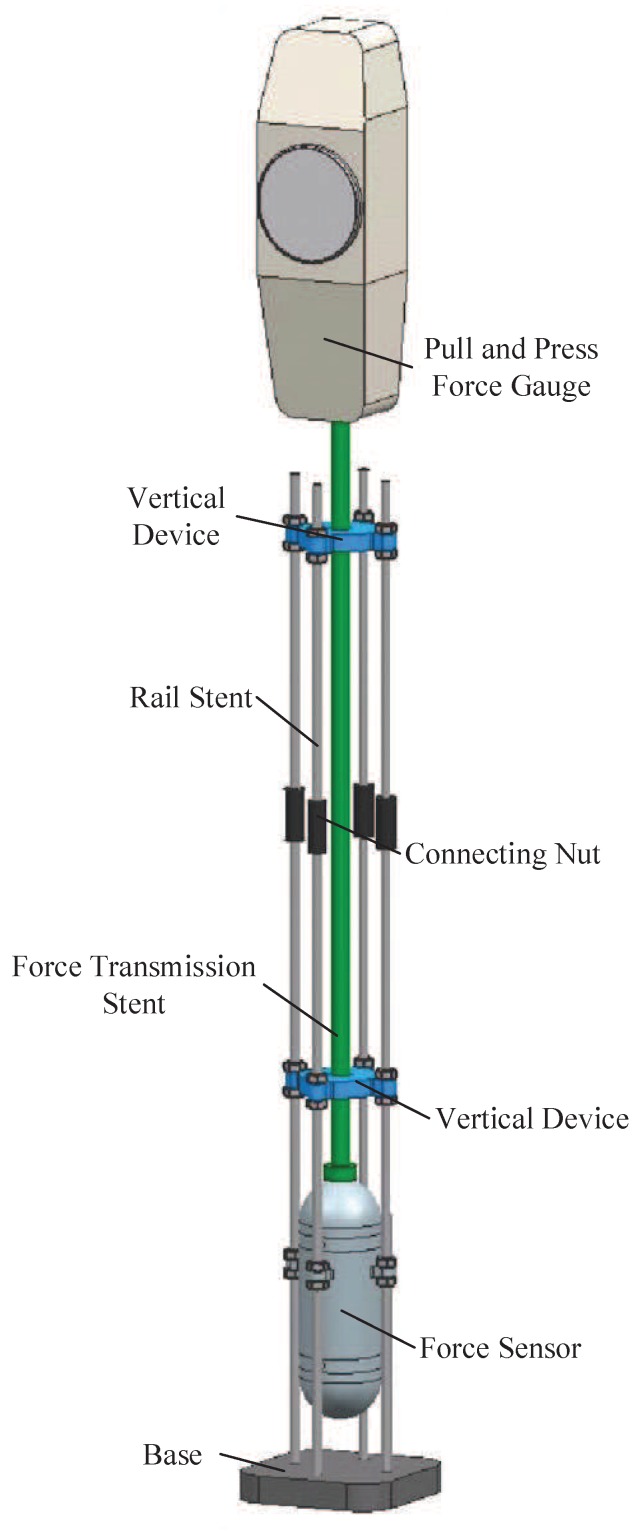
The schematic diagram of underwater standard force loading.

The UTFS generates interactive force for the external grasping target with the hemispherical flexible contact, and an force applying experiment under different water depth is designed for the UTFS output characteristics study. With the capacity of adjustable standard force applying and adjustable water depth supply and also adjustable temperature supply, the UTFS applying device is different from the non-underwater environment one. To realize the application of adjustable tactile force under adjustable water depth, an experimental applying device is designed as shown in Figure 16. Four rail stents are fixedly connected by a vertical device and the extremity is mounted on the base. The UTFS is installed at the end side of the rail stent through the mounting holes and is located above the base in order to achieve the maximum depth of the water environment. The force transmission stent is connected with the pull and press force gauge with the nut and thread, which achieves contact with the upper flexible contact extremity of UTFS through the vertical device to be vertical, ensures the operator apply the force on the pull and press force gauge to the UTFS in the vertical direction contact. To realize the water depth adjustment of the UTFS task environment, and the rail stent can be extended by the connection with another rail stent by the screw thread and the nut, so that the force transmission stent can also be extended with the same way. The operator can do the UTFS experiment at different water depth by operating the experimental device on the surface of the water, realizing the different depths and different tactile force supplies.

**Figure 17:**
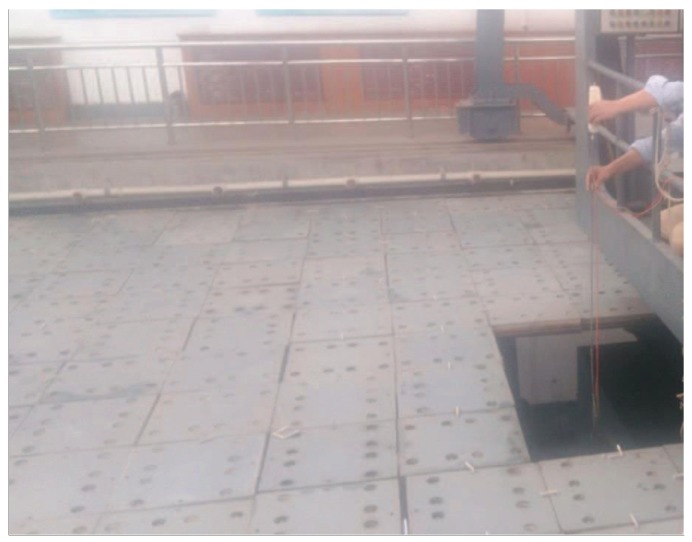
Photo: the pool experiment of UTFS.

**Figure 18:**
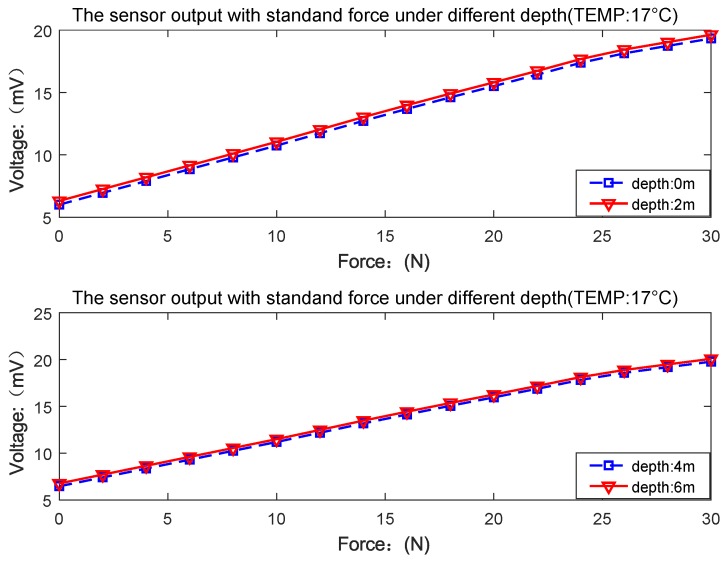
UTFS output with standard force loading at different depths.

In the experimental process of UTFS measurement at any water depth, the sensor is placed in the depth environment of 0 m, 2 m, 4 m and 6 m respectively, and the UTFS is applied with different force to measure the sensor output. The water 0 m depth indicates out of the water, and the water temperature is 17 ${}^{\circ }$C. The pool experiment photo is shown in Figure 17, and the UTFS output curves at different depths are shown in Figure 18.

As shown in Figure 18, the output of the UTFS remains approximately linear with the increase of the standard tactile force signal applied under water depth conditions of 0 m, 2 m, 4 m, and 6 m respectively, and the maximum nonlinear error of the tactile force sensor is 0.21% F.S. The UTFS has the overall linear migration of output value with the increase of water depth affecting the sensor’s zero point drift, and the calibration algorithm can be employed to eliminate the effects of the water depth. Therefore, the UTFS can be data post-processed to avoid the influence of the water pressure, which directly measures the signal of the tactile force. The flexible contact ensures the application of the force signal and the hydraulic balance of the sensor.

### 6.4. UTFS Experiment with Temperature Influence

**Figure 19:**
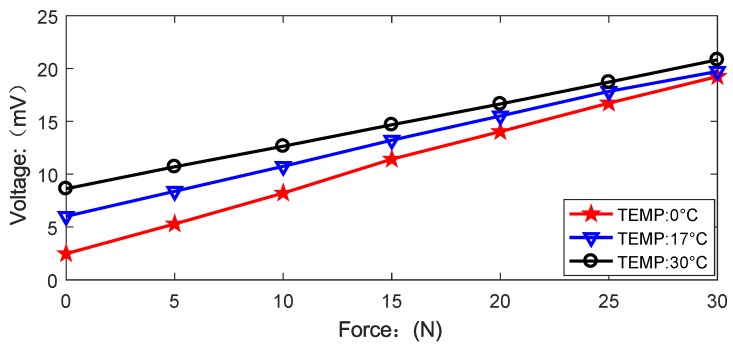
The diagram of UTFS output under different temperature.

The silicon cup sensor core is susceptible to the temperature drift. In order to achieve the reliability and stability of the UTFS to ensure the application of underwater temperature range of 0 ${}^{\circ }$C–30 ${}^{\circ }$C, a temperature test experiment has been done for the designed UTFS output character. The UTFS is placed in the basin with 0.1 m water depth environment, and the water temperature is set to 0 ${}^{\circ }$C, 17 ${}^{\circ }$C, and 30 ${}^{\circ }$C respectively with ice and water mixture or thermal and cool water mixture, and 17 ${}^{\circ }$C water temperature is the indoor natural water temperature. The sensor output is measured under different tactile force load, and the sensor output curves diagram at different temperature is shown in Figure 19.

As can be seen in Figure 19 that the UTFS output is affected by the temperature, and the output with the same tactile force applying is different under different temperature environment. The maximum temperature drift occurs at the zero point, and the UTFS output is shifted from 2.48 mV to 8.63 mV when the water temperature changes from 0 ${}^{\circ }$C to 30 ${}^{\circ }$C. However, the UTFS still maintains good linearity at each fixed temperature point, and the slope of different regression linear with different temperature is different. The temperature influence can be eliminated by the temperature compensation with data-fitting algorithm [47,48].

## 7. Discussion

The main advantage of UTFS is the structure of isolating physical contact with the sensor from water pressure under different water depths. Due to the depth limitation of the pool, the UTFS can only be tested under 6 m depth environment.The UTFS has a 31.5 mm shell length and the flexible contact radius of 25 mm, and realizes the force measurement with sensor core of silicon-core type elastomer under the differential pressure structure. The UTFS maintains the linear output under the condition of the hemispherical flexible contacts interacting with the outside target. The UTFS has the zero drift with different water pressure; however, the force-electric performance output of the sensor linear regression equation on each water pressure point has an almost equal slope. If the accuracy is not high enough in the application, the actual tactile force value can be directly calculated by the zero point output under water pressure and the force applying output. On the contrary, in order to obtain a higher tactile force signal, a backpropagationt (BP) neural network including three input layers consisted force measurement value, temperature measurement value, water pressure measurement value and one output layer consisted the true applied force value may be established, which can be established to calibrate the UTFS by off-line training algorithm to meet the precision tactile force output requirement.

The designed UTFS does not need to attach the strain gauge or to produce a silicon cup type elastomer, which only needs to install the sensor core. The UTFS production reduces the effect of the manufacturing process on the sensor output; however, this kind of structure design method cannot realize the high density array distribution of underwater robot dexterous hand because of the big volume restriction. The silicon cup elastomer of the sensor core can be directly mounted on the dexterous hand finger to realize the miniaturization of force measurement. In the measurement experiment, the UTFS output has a higher resolution and sensitivity character when the applying force point is the vertices of hemispherical flexible contact and vertical contact or the output will be biased if the applying force on the side of the hemisphere flexible contact.

The UTFS’s nonlinear error, repeatability and hysteresis are respectively 0.21% F.S, 0.02% F.S, 0.02% F.S, and the UTFS’s sensitivity is 0.47 mV/N, which is priority to Yong xin Guo’s force sensor [49]. The tactile force sensor has a reasonable structure and is easy to assemble. The full arm Wheatstone bridge achieves high sensitivity of the output signal and can be well applied to underwater tactile force measurement. Compared with Qinxin’s work [16], it reduces the complexity of the circuit, and it save the structural design cost compared to Yi’s fiber optic tactile force sensor [19]. The main experiment is under the still water to measure the character of the sensor, and the water fluctuation may affect the sensor output when considering the complexity of seawater environment. Because the hemispherical flexible contact realizes the isolation of the external seawater from the internal silicone oil and the tactile force signal will have a small threshold value, which will have a certain effect on the sensor when the seawater affection is more than the threshold for the sensor information. The sensor can also be used to achieve underwater object properties judgment.

## 8. Conclusions

In this paper, the UTFS used in the array distribution of underwater dexterous hand to realize tactile force measurement and grasping position determination is analyzed. The capsule structure of UTFS is introduced, which realizes the vector superposition of water static pressure on the upper and lower side of the sensor core to achieve the tactile force measurement with the influence of water pressure eliminated. The stress and strain character of silicon cup elastomer is analyzed and verified by Finite Element Analysis to support the strain element distribution on the surface of silicon cup. To verify the performance of the designed UTFS, the zero point output at different depth experiment, the different water depth arbitrary force applying experiment, and the temperature influence output experiment were implemented under the condition of 6 m water depth environment. The results show that: The structure of the capsule shape with differential pressure technology makes the UTFS to eliminate the water static pressure. The silicon cup measurement structure satisfies the characteristics of high sensitivity and large output stress. The error of the tactile force measurement sensor is small and the linearity is high. If the designed sensor array is distributed on the finger of the proposed underwater dexterous hand, it is easy to determine the grasping position of the underwater target.

## Figures and Tables

**Table 1:** The selected sensor core parameters.

Item	Nonlinear	Repeatability	Hysteresis	Zero Output	Full-Scale Output
Typical value	±0.15	±0.05	±0.05	±2	
Maximum	±0.25	±0.075	±0.075		
Minimum					45
Unit	%FS, BFSL	%FS	%FS	mV DC	mV DC
